# Association between mesothelin expression and survival outcomes in patients with triple-negative breast cancer: a protocol for a systematic review

**DOI:** 10.1186/s13643-016-0313-6

**Published:** 2016-08-11

**Authors:** Mei Wang, Aihua Li, Guangwen Sun, Lawrence Mbuagbaw, Susan Reid, Peter J. Lovrics, Lehana Thabane

**Affiliations:** 1 Department of Clinical Epidemiology and Biostatistics, McMaster University, 1280 Main Street West, Hamilton, L8S 4K1 Ontario Canada; 2 Biostatistics Unit, Father Sean O’Sullivan Research Centre, St. Joseph’s Healthcare, 3rd floor Martha Wing, 50 Charlton Avenue East, Hamilton, L8N 4A6 ON Canada; 3 Centre for Development of Best Practices in Health, Yaoundé Central Hospital, Yaoundé, Cameroon; 4 Department of Pediatrics and Anesthesia, McMaster University, Hamilton, Canada; 5 Centre for Evaluation of Medicine, St Joseph’s Healthcare Hamilton, Hamilton, Canada; 6 Hamilton Health Sciences, Population Health Research Institute, Hamilton, Canada; 7 Dundas Valley Secondary School, Hamilton, Canada; 8 General Surgery, Juravinski Hospital, 711 Concession St. E, Hamilton, L8V 1C3 ON Canada; 9 Department of Surgery, Surgical Outcomes Research Centre, McMaster University and St. Joseph’s Healthcare, 50 Charlton Avenue East, Hamilton, ON L8N 4A6 Canada

**Keywords:** Triple-negative breast cancer, Mesothelin, Meta-analysis, Survival, Systematic review

## Abstract

**Background:**

Mesothelin is a membrane-bound glycoprotein. Although the biologic function of mesothelin is not very clear, researchers have found that it plays a role in the survival, proliferation, and migration of tumor cells. Identified as a tumor-associated biomarker, mesothelin is more often overexpressed in triple-negative breast cancer (TNBC) than in common luminal breast tumor subtype or normal tissues. The objective of this review is to determine the association between the expression of mesothelin and overall survival in patients with TNBC.

**Methods/design:**

We will search the following electronic databases: MEDLINE, EMBASE, the Cochrane Central Register of Controlled Trials (CENTRAL), PubMed, and Web of Science with no time or language restriction. Prospective or retrospective longitudinal studies that investigate mesothelin expression in TNBC or the prognosis of TNBC with mesothelin baseline measurement will be selected. Two reviewers will independently assess every abstract or full text for inclusion. Data on clinical outcomes, as well as on study design, research setting, study population, demographic characteristics of the participants, and methodological quality, will be extracted using a structured codebook developed by the authors. A pooled measure of associations will be assessed through meta-analyses if appropriate. Heterogeneity across the included studies will be evaluated using the *I*^2^ statistics. Findings will be reported according to the Meta-Analysis of Observational Studies in Epidemiology (MOOSE) guidelines and the Preferred Reporting Items for Systematic Reviews and Meta-Analyses (PRISMA) statement. The quality of evidence and risk of bias of the studies will be evaluated.

**Discussion:**

The aim of this systematic review is to synthesize the evidence regarding the association between the expression of mesothelin and the survival outcomes of patients with TNBC. A better understanding of the expression frequency and prognostic value of mesothelin in TNBC will be essential to identifying a novel therapeutic target.

**Systematic review registration:**

PROSPERO CRD42016036212

**Electronic supplementary material:**

The online version of this article (doi:10.1186/s13643-016-0313-6) contains supplementary material, which is available to authorized users.

## Background

### Description of the condition

Triple-negative breast cancer (TNBC), which is characterized by a lack of expression of estrogen receptor (ER), progesterone receptor (PR), and human epidermal growth factor receptor 2 (HER2), is an aggressive subtype of breast neoplasms. About 15 % of breast cancers are TNBC [1], and they tend to be more common in younger women [2]. TNBC has a relatively higher rate of relapse and worse overall survival rate than other forms of breast cancer [3].

### Description of the prognostic factor

Mesothelin is a membrane-bound glycoprotein. Its expression is limited to mesothelial cells and some malignant neoplasms [4]. The biological function of mesothelin is not very clear. However, researchers have found that it has a role in the survival, proliferation, and migration of tumor cells [5].

### How the prognostic factor may be related to health outcomes

Mesothelin is identified as a biomarker for TNBC because it is overexpressed in TNBC while its expression is limited in the common luminal breast tumor subtype and normal tissues [6]. As such, it is hypothesized that the expression of mesothelin in TNBC is of relevance for patients’ outcome.

Although TNBC is more sensitive to chemotherapy than other types of breast cancer, cytotoxic agents often result in numerous adverse side effects. Targeted therapies on specifically overexpressed antigens have been successful in the treatment of some malignant diseases [7]. TNBC lacks effective targeted therapies that can improve its chemotherapy. Mesothelin is a potential candidate for targeted therapies for TNBC because of its overexpression in TNBC cells and limited expression in normal tissues. Therefore, a better understanding of the expression levels of mesothelin and its prognostic value in TNBC may provide evidence necessitating the role of mesothelin as a therapeutic target, thus leading the path of improving health outcomes of patients with TNBC.

### Why it is important to do this review

Li et al. and Tozbikian et al. showed that the levels of mesothelin expression in TNBC were an independent prognostic marker that correlates with low survival rates [8, 9]. However, two other studies failed to demonstrate such an association between the presence of mesothelin and poor clinical outcome [10, 11]. These controversial results may result from insufficient samples and different study designs. To the best of our knowledge, no systematic review or meta-analysis has ever been done on this topic. The present systematic review aims to evaluate the frequency of mesothelin expression and the value of mesothelin as a prognostic marker for TNBC.

## Objectives

The primary objective of this review is to synthesize available evidence on the association between the expression of mesothelin and overall survival of patients with TNBC. The secondary objectives include determining the association between the expression of mesothelin and disease-free survival (DFS), relapse-free survival (RFS), distant metastases, and mortality and, where feasible, combining the results of original studies using meta-analysis and critically evaluating the literature and identifying future research needs.

## Methods

### Study registration

This project has been registered with the international prospective register of systematic reviews (PROSPERO) as number CRD42016036212. This systematic review protocol has been designed and reported using Preferred Reporting Items for Systematic Review and Meta-Analysis Protocols (PRISMA-P) 2015 statement [12] and the Meta-Analysis of Observational Studies in Epidemiology (MOOSE) guidelines [13]. The PRISMA-P checklist has been used in the preparation of this protocol and is included as an additional file (see Additional file 1).

### Eligibility criteria

#### Types of study designs

We will include both prospective and retrospective longitudinal studies in this systematic review. The studies will be eligible if they evaluated mesothelin expression in TNBC or the prognosis of TNBC with mesothelin at baseline measurement. We will consider both exploratory and confirmatory prognostic factor investigation studies. When overlapping data of the same patient population are included in more than one publication, only the most recent or complete study will be used in the meta-analysis.

#### Types of participants and setting

Studies would be included in this systematic review if the participants had TNBC. No age, intervention, or setting restrictions will be placed.

#### Exposure variable

For the prognostic studies, our main exposure will be mesothelin expression measured by immunohistochemical analysis.

#### Types of outcome measures

Our primary outcome will be overall survival (OS). If the OS is not reported in the study, we will collect its relevant surrogate outcomes, which include DFS or RFS, distant metastases, and mortality.

#### Time frame

The prognostic studies should be at least 1-year duration for follow-up. We will group outcome data into three time periods for analysis purposes: short-term (≤3 years), medium-term (3–5 years), and long-term follow-up (≥5 years).

### Hypothesis

The research hypothesis of this systematic review is that the expression of mesothelin in patients with TNBC is associated with poor health outcomes.

- Primary outcome
  - The primary outcome of interest is the OS in patients with mesothelin-positive TNBC, which were estimated from the date of surgery to the date of the final follow-up visit, or the date of the end of the study, or the date of death.
- Secondary outcomes
  - DFS and RFS—time-to-event data which were measured from the date of surgery to the date of the first documented local or distant recurrence. Patients who died or lost to follow-up before experiencing the relevant events were considered censored at their dates of the last follow-up.
  - Distant metastases—estimated by subsequent distant metastases, interval to metastases.
  - Mortality—measured by mortality rate.

### Search methods for identification of studies

#### Electronic searches

A comprehensive literature search will be done for original articles analyzing the expression and prognostic value of mesothelin in TNBC. The electronic database sources we will search are MEDLINE, EMBASE, the Cochrane Central Register of Controlled Trials (CENTRAL), PubMed, and Web of Science with no restrictions on time and languages. As there may be limited published research on mesothelin in TNBC, we will increase our search sensitivity by finding any articles related to TNBC and mesothelin. The search terms will be “triple negative breast cancer,” “triple negative breast carcinoma,” “triple negative breast neoplasm,” “TNBC,” and “mesothelin.”

A three-step search strategy will be utilized in this review. First, we will explore database subject headings and text words. An initial pilot search in MEDLINE will be undertaken followed by analysis of the text words contained in the title and abstract and of the subject headings used to describe the article. Second, we will search using all identified keywords and index terms across all included databases. And finally, the reference list of all identified reports and articles will be searched for additional studies. Studies published in all languages will be considered for inclusion in this review. Please refer to Additional file 2 for the full search strategy and Additional file 3 for the primary literature screening form we will use during this stage.

#### Searching other resources

We will search for additional references by cross-checking bibliographies of retrieved studies or relevant reviews. We will also contact researchers in the field to identify additional trials that may have been eligible for inclusion. Mediconf (http://www.mediconf.com/) will be used to search conference abstracts.

### Data collection and analysis

#### Selection of studies

Our selection of studies will follow the four-phase flow diagram (Additional file 4) referring to the PRISMA statement. The literature search results will be uploaded to EndNote X7 Software, which will facilitate the collaboration among reviewers during the study selection process.

The screening will be conducted using a pretested screening form. Titles and abstracts will first be screened for relevance and presence of the eligible criteria listed above (the second phase). Full-text articles for potentially eligible titles or abstracts will be downloaded for further assessment. Articles will be classified as (1) included, (2) excluded, or (3) uncertain. For each phase of screening, the kappa statistic will be performed to calculate inter-rater agreement. Two authors (MW and AL) will independently perform the filtering of the selected pieces of literature. Disagreements will be resolved by consensus or by contacting a third author when consensus cannot be reached (LM or LT).

#### Data extraction and management

Data will be collected using a predesigned data collection form which will be pilot tested beforehand (Additional file 5). The following details will be extracted: study characteristics (such as the first author, the country, the year of publication, the study design, and the sample size); participants’ characteristics (the age, the stage of breast cancer, and the interventions); study exposure (the technique used to quantify mesothelin, the cut-off to determine mesothelin positivity, and the number of mesothelin positivity and controls); and outcome variables (median OS, median disease-free survival (DFS) or relapse-free survival (RFS), number of distant metastases, number of deaths, and 95 % confidence intervals, as well as events rates and numbers at risk during short-term (≤3 years), medium-term (3–5 years), and long-term follow-up (>5 years more) intervals). If a study has insufficient details to extract the number of events, hazard ratio (95 % CI) or odds ratio (95 % CI), but provides the survival curves, we will use the survival curve approach to extract required data to obtain estimates of association [15].

If necessary, modifications will be done to the data collection form after testing the reliability of data abstraction on a random sample. The review authors (WM and AL) will independently extract the data, resolve discrepancies by discussion, or refer to the third reviewer (LM or LT).

Breast carcinoma was defined as TNBC when nuclear staining is <1 % for both ER and PR, and membranous staining is <10 % of HER2 on immunohistochemistry (IHC) staining or negative gene amplification was found by fluorescence in situ hybridization (FISH) [16, 17]. Generally, an H-score is used to quantify the mesothelin staining. H-score (final score ranging between 0 and 300) is the combination of the percentage of tumor cells with positive staining (0–100 %) and the intensity of staining (1+, 2+, and 3+) [18]. We will choose H-score ≤10 as mesothelin-negative and H-score >10 as mesothelin-positive [18]. The survival data will be extracted from the tables or texts of eligible articles or estimated from Kaplan-Meier curves where it is applicable [19]. If we encounter any missing or unclearness of above information, study authors will be contacted for additional details.

#### Quality assessment of included studies

The Newcastle-Ottawa Assessment Scale (NOS) [18] will be employed by two independent reviewers (MW and AL) to assess the methodological quality of all eligible trials. The adapted version of the NOS will be used to meet the specific needs of this systematic review [20]. Included studies will be assessed on three broad perspectives: the selection of the study groups, the comparability of the groups, and the ascertainment of either the exposure or outcome of interest for case-control or cohort studies, respectively [20]. Reviewers will classify the risk of biases in the studies on a scale from 0 to 3, where 0 indicates a high risk of bias and 3 indicates low risk. The adapted version of the NOS includes seven questions spread across four domains of evaluation: methods for selecting study participants (selection bias), methods to control for confounding (performance bias), statistical methods (detection bias), and methods for measuring exposure and outcome variables (information bias). These scales will be used to measure the risk of bias on a per study basis or categorized by domain to develop a general conclusion about the sources of bias in the studies included in this review (see Additional file 6) [21]. Reviewers will resolve disagreements by discussion, and one arbitrator will adjudicate unresolved disputes (LM or LT).

#### Data synthesis and statistical analysis

The statistical reporting will be performed according to the guidelines proposed by the MOOSE group [22]. Descriptive statistics will be used to describe the characteristics of all eligible studies. Data will be presented as mean (standard deviation) or median (first quartile, third quartile) for both continuous variables and frequencies (percent) for categorical variables. If there are a sufficient number of studies (at least three) suitable for pooling, we will perform meta-analyses of the studies to obtain a pooled estimate of the association between the expression levels of mesothelin and the survival of TNBC expressed as odds ratio (at similar follow-up points) or hazard ratio with 95 % confidence intervals (CIs). This analysis will be conducted using STATA. When heterogeneity is present, a random effect model (Der Simonian and Laird method) will be applied, while the fixed effect model will be used in the absence of between-study heterogeneity (*P* > 0.10 or *I*^2^ < 50 %) [23]. We will consider the clinical heterogeneity of included studies based on outcome measurements, follow-up length, and methodological heterogeneity due to study design (prospective versus retrospective).

We will synthesize data within these clinically relevant subgroups. Between-study heterogeneity will be measured using the Cochrane’s Q test and Higgins *I*^2^ statistic (*P* value of <0.10 or *I*^2^ > 50 % will be considered as statistically significant heterogeneity). If there is a large number studies, we will use meta-regression or subgroup analyses to explain the heterogeneity by lymph node metastasis, pathological stage, and adjuvant chemotherapy.

#### Dealing with missing participant data, sensitivity analyses, and publication bias

For missing data, we will contact study authors for clarification and to attempt to retrieve any missing information. We will conduct a complete case analysis (excluding those with missing data) as the primary analysis [24], and a sensitivity analysis will be performed to determine the articles with more than 25 % missing data which influence the overall result. If feasible, we will also perform a sensitivity analysis by excluding trials with a high risk of bias to test the robustness of our results.

If the number of eligible studies is too small (<10), publication bias will not be assessed. If there are 10 or more eligible studies, publication bias will be examined for each meta-analysis by visually examining asymmetry of funnel plots and testing for asymmetry at the 10 % level, using Egger’s test for hazard ratios and Peters’ test for odds ratios [25].

## Discussion

The lack of expression of conventional prognostic markers (estrogen, progesterone, and Her2 receptors) in TNBC denies those patients the benefit of targeted therapy against these receptors. The search for a molecular therapeutic target for TNBC is ongoing. Mesothelin is identified as a biomarker for TNBC because some authors have identified its overexpression in TNBC and limited expression in the common luminal breast tumor subtype and normal tissues [6]. A better understanding of the expression frequency and prognostic value of mesothelin in TNBC will be essential to identify a novel therapeutic target with the goal of improving health outcomes of patients with TNBC.

This proposed review will serve several purposes. First, we hope to identify the expression level of mesothelin in TNBC. Second, we will document survival rates linked to the levels of mesothelin in TNBC. Our results will inform disease prognosis and highlight better therapeutic and diagnostic timing strategies. There are ongoing investigations of agents that target mesothelin-expressing tumors. Even if we find mesothelin has little prognostic value in patients with TNBC, it may still be a promising target for novel drug therapies in other cancers. Finally, we hope the results of this review will encourage future research in identifying mesothelin as a novel target for improving the outcomes for patients with TNBC.

Our systematic review will be conducted according to recommended standards including explicit eligibility criteria, duplicate independent assessment of eligibility, and a comprehensive search. We will use the NOS approach to assessing methodological quality including duplicate independent assessment of the validity of the individual studies. Our results are only likely to be restricted by limitations in the primary studies. To our knowledge, this review will be the first systematic review about the association between the expression levels of mesothelin and clinical outcomes in patients with TNBC.

## Abbreviations

AL, Aihua Li; DFS, disease-free survival; ER, estrogen receptor; GRADE, Grading of Recommendations, Assessment, Development and Evaluation; GS, Guangwen Sun; HER2, human epidermal growth factor receptor 2; LM, Lawrence Mbuagbaw; LT, Lehana Thabane; MW, Mei Wang; OS, overall survival; PL, Peter Lovrics; PR, progesterone receptor; SR, Susan Reid; TNBC, triple-negative breast cancer

## Additional files

Additional file 1: Preferred Reporting Items for Systematic review and Meta-Analysis Protocols (PRISMA-P) 2015 checklist for “Association between mesothelin expression and survival outcomes in patients with triple-negative breast cancer: a protocol for a systematic review” [12]. (DOC 80 kb) Additional file 2: Search strategies in MEDLINE, EMBASE, Cochrane Central Register of Controlled Trials, and web of science databases. (DOCX 12 kb) Additional file 3: Primary literature screening form. (DOCX 12 kb) Additional file 4: PRISMA flow diagram [14]. (DOCX 98 kb) Additional file 5: Data extraction form. (DOCX 12 kb) Additional file 6: Modified Newcastle-Ottawa Scale (NOS) (Bawor M. et al.). (DOCX 14 kb)
