# Supplementary material for: Association between mesothelin expression and survival outcomes in patients with triple-negative breast cancer: a protocol for a systematic review
Source: Syst Rev. 2016 Aug 11;5:133. doi: 10.1186/s13643-016-0313-6 (PMC4982336; doi:10.1186/s13643-016-0313-6)
Supplement: Additional file 2: — Search strategies in MEDLINE, EMBASE, Cochrane Central Register of Controlled Trials, and web of science databases. (DOCX 12 kb) [file 13643_2016_313_MOESM2_ESM.docx]

**Appendix 1. Search strategies in MEDLINE, EMBASE and Cochrane Central Register of Controlled Trials, and web of science databases**

| Database: Ovid MEDLINE(R) In-Process & Other Non-Indexed Citations, Ovid MEDLINE(R) Daily and Ovid MEDLINE(R) <1946 to Present>  Search Strategy:  --------------------------------------------------------------------------------  1 ((Triple$ or triple negative) adj6 (breast cancer$ or breast neoplasm$ or breast tumo$ or carcinoma$)).mp. [mp=title, abstract, original title, name of substance word, subject heading word, keyword heading word, protocol supplementary concept word, rare disease supplementary concept word, unique identifier] (3999)  2 exp Membrane Glycoproteins/ or exp GPI-Linked Proteins/ or mesothelin.mp. (553957)  3 1 and 2 (181) |
| --- |
| Database: Embase <1974 to present>  Search Strategy:  --------------------------------------------------------------------------------  1 ((Triple$ or triple negative) adj6 (breast cancer$ or breast neoplasm$ or breast tumo$ or carcinoma$)).mp. [mp=title, abstract, heading word, drug trade name, original title, device manufacturer, drug manufacturer, device trade name, keyword] (9404)  2 mesothelin.mp. or exp mesothelin/ (1282)  3 1 and 2 (17) |
| Cochrane search results: 0 article |
| Database: web of science  # 1 799 TOPIC: (mesothelin)  Indexes=SCI-EXPANDED, SSCI, A&HCI, CPCI-S, CPCI-SSH, ESCI Timespan=All years  # 2 14 TOPIC: (mesothelin)  Refined by: TOPIC: (triple negative breast)  Indexes=SCI-EXPANDED, SSCI, A&HCI, CPCI-S, CPCI-SSH, ESCI Timespan=All years |
| Database: PubMed  #1 “mesothelin"[Supplementary Concept] OR "mesothelin"[All Fields] (702)  #2 "triple negative breast neoplasms"[MeSH Terms] OR ("triple"[All Fields] AND "negative"[All Fields] AND "breast"[All Fields] AND "neoplasms"[All Fields]) OR "triple negative breast neoplasms"[All Fields] OR ("triple"[All Fields] AND "negative"[All Fields] AND "breast"[All Fields] AND "cancer"[All Fields]) OR "triple negative breast cancer"[All Fields] (5238)  #1 and #3 (8) |
