# Supplementary material for: Association between mesothelin expression and survival outcomes in patients with triple-negative breast cancer: a protocol for a systematic review
Source: Syst Rev. 2016 Aug 11;5:133. doi: 10.1186/s13643-016-0313-6 (PMC4982336; doi:10.1186/s13643-016-0313-6)
Supplement: Additional file 3: — Primary literature screening form. (DOCX 12 kb) [file 13643_2016_313_MOESM3_ESM.docx]

**Appendix 2 (table): Primary literature screening form**

| First author, Year: abstractor initials: | | | |
| --- | --- | --- | --- |
| Eligible criteria | | | |
| 1. Types of study designs: Measures the relationship between mesothelin expression and an outcome (i.e., not a descriptive study), or measures the mesothelin expression level in TNBC. | Yes | No | Not sure |
| 2. Reports original research. NOTE: If the publication appears relevant to the topic, consider whether it should be retained for “review for references”. | Yes | No | Not sure |
| 3. Population is patients with triple negative breast cancer. | Yes | No | Not sure |
| 4. Exposure variable: For the prognostic studies, our main exposure is mesothelin expression measured by immunohistochemically analysis. |  |  |  |
| 5. Types of outcome measures: primary outcome: OS. Relevant surrogate outcomes, which include Disease-free survival (DFS) or relapse-free survival (RFS), distant metastases, and mortality. |  |  |  |
| 6. Time frame: follow up time ≥3 years | Yes | No | Not sure |
| Retain for:  □ Background/Discussion □ Review of references □ Harms data □ Other_____________  COMMENTS: | | | |
