# Supplementary figures and images for: Association between mesothelin expression and survival outcomes in patients with triple-negative breast cancer: a protocol for a systematic review
Source: Syst Rev. 2016 Aug 11;5:133. doi: 10.1186/s13643-016-0313-6 (PMC4982336; doi:10.1186/s13643-016-0313-6)

**Figure 3.** PRISMA flow diagram14.


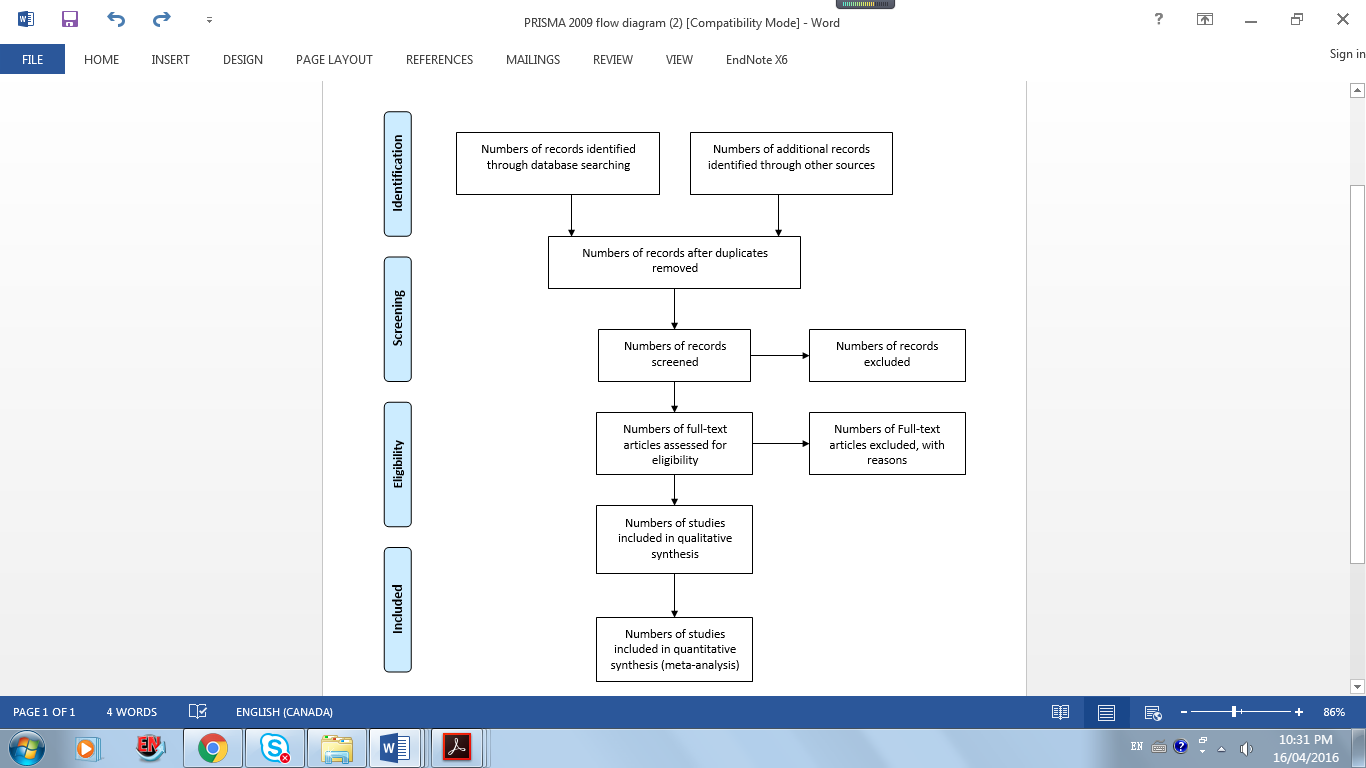

Supplement: Additional file 4: — PRISMA flow diagram [14]. (DOCX 98 kb) [file 13643_2016_313_MOESM4_ESM.docx]
