# Supplementary material for: Association between mesothelin expression and survival outcomes in patients with triple-negative breast cancer: a protocol for a systematic review
Source: Syst Rev. 2016 Aug 11;5:133. doi: 10.1186/s13643-016-0313-6 (PMC4982336; doi:10.1186/s13643-016-0313-6)
Supplement: Additional file 5: — Data extraction form. (DOCX 12 kb) [file 13643_2016_313_MOESM5_ESM.docx]

**Appendix 4. Data extraction form**

Study ID: ______ Reviewer Initials: _______

**STUDY INFORMATION**

First Author: ____________________ Year of Publication__________

Title of Article: _________________________________________________________________

Journal Name: _______________________________ Country: ____________________

**METHODS**

Study Setting: _____________________________ Study Design: _______________________

Sample Size: Total________. The number of mesothelin (+) ______________and the # of mesothelin (-) ________________.

Mean Age (SD): ______, the stage of breast cancer__________________, and the interventions_________________________________________________.

The technique used to quantify mesothelin______________________________________________________. The cut-off to determine mesothelin positivity_________________________________________________

**RESULTS (survival data)**

|  | Positive mesothelin expression | | | | Negative mesothelin expression | | | | Total |
| --- | --- | --- | --- | --- | --- | --- | --- | --- | --- |
|  | total | ≤3 years | 3-5 years | >5 years | total | ≤3 years | 3-5 years | >5 years |  |
| Sample size |  | | | |  | | | |  |
| Median overall survival (OS)  (95%CI) |  |  |  |  |  |  |  |  |  |
| Median disease-free survival (DFS)  (95%CI) |  |  |  |  |  |  |  |  |  |
| number of distant metastases |  |  |  |  |  |  |  |  |  |
| number of deaths |  |  |  |  |  |  |  |  |  |

COMMENTS

______________________________________________________________________________

_____________________________________________________________________________
