# Supplementary material for: Association between mesothelin expression and survival outcomes in patients with triple-negative breast cancer: a protocol for a systematic review
Source: Syst Rev. 2016 Aug 11;5:133. doi: 10.1186/s13643-016-0313-6 (PMC4982336; doi:10.1186/s13643-016-0313-6)
Supplement: Additional file 6: — Modified Newcastle-Ottawa Scale (NOS) (Bawor M. et al.). (DOCX 14 kb) [file 13643_2016_313_MOESM6_ESM.docx]

**Appendix 5. Modified Newcastle-Ottawa Scale (NOS) (Bawor M. et al)**

Legend

0 = definitely no (high risk of bias) 1 = Mostly no

2 = Mostly yes 3 = definitely yes (low risk of bias)

**Domain of evaluation: Methods for selecting study participants (i.e. Selection bias)**

Is the source population (cases, controls, cohorts) appropriate and representative of the population of interest? (0 1 2 3)

Is the sample size adequate and is there sufficient power to detect a meaningful difference in the outcome of interest? (0 1 2 3)

**Domain of evaluation: Methods to control confounding (i.e. Performance bias)**

Did the study identify and adjust for any variables or confounders that may influence the outcome?

(0 1 2 3)

Did the study use appropriate statistical analysis methods relative to the outcome of interest?

(0 1 2 3)

Is there little missing data and did the study handle it accordingly?

(0 1 2 3)

**Domain of evaluation: Statistical methods (i.e. Detection bias)**

Is the methodology of the outcome measurement explicitly stated and is it appropriate?

(0 1 2 3)

Is there an objective assessment of the outcome of interest?

(0 1 2 3)
